# Cardiothoracic imaging findings of Proteus syndrome

**DOI:** 10.1038/s41598-021-86029-0

**Published:** 2021-03-22

**Authors:** S. Mojdeh Mirmomen, Andrew E. Arai, Evrim B. Turkbey, Andrew J. Bradley, Julie C. Sapp, Leslie G. Biesecker, Arlene Sirajuddin

**Affiliations:** 1 Cardiovascular and Pulmonary Branch, National Heart Lung and Blood Institute, National Institutes of Health, Building 10, Room B1D416, 10 Center Drive, Bethesda, MD 20814 USA; 2 Radiology and Imaging Sciences, National Institutes of Health, Building 10, Room 1C336, Bethesda, MD 20814 USA; 3 Genetic Disease Research Branch, National Human Genome Research Institute, National Institutes of Health, Building 10, Room 8D47E, Bethesda, MD 20814 USA

**Keywords:** Cardiovascular diseases, Respiratory tract diseases, Genetics, Diseases

## Abstract

In this work, we sought to delineate the prevalence of cardiothoracic imaging findings of Proteus syndrome in a large cohort at our institution. Of 53 individuals with a confirmed diagnosis of Proteus syndrome at our institution from 10/2001 to 10/2019, 38 individuals (men, n = 23; average age = 24 years) underwent cardiothoracic imaging (routine chest CT, CT pulmonary angiography and/or cardiac MRI). All studies were retrospectively and independently reviewed by two fellowship-trained cardiothoracic readers. Disagreements were resolved by consensus. Differences between variables were analyzed via parametric and nonparametric tests based on the normality of the distribution. The cardiothoracic findings of Proteus syndrome were diverse, but several were much more common and included: scoliosis from bony overgrowth (94%), pulmonary venous dilation (62%), band-like areas of lung scarring (56%), and hyperlucent lung parenchyma (50%). In addition, of 20 individuals who underwent cardiac MRI, 9/20 (45%) had intramyocardial fat, mostly involving the endocardial surface of the left ventricular septal wall. There was no statistically significant difference among the functional cardiac parameters between individuals with and without intramyocardial fat. Only one individual with intramyocardial fat had mildly decreased function (LVEF = 53%), while all others had normal ejection fraction.

## Introduction

Proteus syndrome is a rare post-natal overgrowth disorder with asymmetric overgrowth of any body tissue. It has a mosaic distribution, progressive course, and sporadic occurrence^1–3^. It was originally described by Temtamy and Rogers in 1976^4^ and named “Proteus syndrome” by Wiedemann et al. in 1983^5,6^. Proteus syndrome is more common in males (male-to-female ratio of 2:1) and has an estimated prevalence of one per one million births^1,3,7,8^. Proteus syndrome is associated with a mosaic, somatic activating variant in *AKT1* (typically c.49G >A p.Glu17Lys) which is an important aid in diagnosis^3,5,9,10^. However, due to the mosaic pattern of its heterogeneous manifestations, adherence to recently revised score-based diagnostic criteria for phenotypic features of Proteus syndrome is useful (Table 1)^9,11^. Proteus syndrome is defined as a score ≥ 10 points with an *AKT1* pathogenic variant or a score of ≥ 15 points without the *AKT1* variant. Individuals who carry *AKT1* pathogenic variant and score 2–9 based on the revised diagnostic criteria are classified as *AKT1*-related overgrowth spectrum^9^.

**Table 1:** Clinical diagnostic criteria of Proteus syndrome^11^.

Positive clinical criteria	Cerebriform connective tissue nevus		+ 5
	Asymmetric, disproportionate overgrowth (one or more)	Limbs	+ 5
		Hyperostosis of the skull	
		Hyperostosis of the external auditory canal	
		Megaspondylodysplasia, scoliosis, or rib hyperostosis	
	Organ/visceral overgrowth (two or more)	Central nervous system	+ 5
		Urogenital system	
		Eye	
		Spleen	
		Kidney	
		Liver	
		Tonsils or adenoids	
		Gingiva or tongue	
	Bullae or cysts of the lungs		+ 2
	Dysregulated adipose tissue (one or more)	Lipomas	+ 2
		Lipodystrophy	
		Myocardial septal lipoma	
	Linear verrucous epidermal nevus		+ 2
	Vascular malformations (one or more)	Capillary malformation	+ 2
		Venous malformation	
		Lymphatic malformation	
	Specific tumors	Female genitourinary cystadenoma (< 11 yo)	+ 1
		Parotid monomorphic adenoma (> 11 yo)	
		Meningioma (meningothelial and transitional subtype)	
		Testicular cystadenomas or cystadenocarcinomas	
	Facial phenotype (three or more features)	Dolichocephaly	+ 2
		Long face	
		Down slanting palpebral fissures and/or minor ptosis	
		Low nasal bridge	
		Wide or anteverted nares	
		Open mouth at rest	
	Deep vein thrombosis and/or pulmonary embolism		+ 2
Negative clinical criteria	Substantial prenatal extracranial overgrowth		− 5
	Ballooning overgrowth		− 5

Proteus syndrome: a score of 10 or more points with a mosaic *AKT1* variant or 15 or more points without an *AKT1* mosaic variant. *AKT1*-related overgrowth spectrum: a score of 2–9 with an *AKT1* mosaic variant.

Cardiothoracic findings in Proteus syndrome are common and can affect the heart, skeleton, adipose tissue of the chest wall and mediastinum, lungs, pleura, vascular structures, and the thymus. Individuals with Proteus syndrome have an increased risk of premature death due to deep vein thrombosis (DVT), pulmonary thromboembolism, pneumonia, and respiratory failure^2,3,5,8,10^. They are also at an increased risk of developing malignancy of any kind, most commonly meningiomas and gonadal tumors^1–3,6,7^.

Although the cardiothoracic radiological manifestations of Proteus syndrome have been described in previous clinical reports^12–15^ and small clinical series^16–18^, to our knowledge, a comprehensive study systematically examining cardiothoracic imaging findings in a large series of individuals has not been reported. Our aim was to systematically evaluate the cardiothoracic imaging manifestations of Proteus syndrome in a large cohort of individuals to better determine their prevalence.

## Materials and methods

### Participant selection

This was a retrospective study analyzing cardiothoracic imaging findings in a cohort of individuals with Proteus syndrome from 10/2001 to 10/2019 (clinicaltrials.gov identifier: NCT00001403). This study was approved by the institutional review board at the National Human Genome Research Institute (NHGRI), Bethesda, MD, USA and was compliant with the Privacy Act (comparable to HIPAA). Individuals were included in this study if they met the following criteria: (1) had a clinical diagnosis of Proteus syndrome and (2) had cross-sectional imaging of the thorax (CT/CTA chest and/or cardiac MRI) available for review. Clinical diagnosis of Proteus syndrome was determined by both the presence of a mosaic AKT1 pathogenic variant as well as meeting clinical diagnostic criteria for Proteus syndrome detailed in Table 1. Individuals who lacked a mosaic AKT1 pathogenic variant or who only met clinical diagnostic criteria for AKT1-related overgrowth spectrum were excluded. Individuals who were clinically diagnosed with Proteus syndrome, but did not have cross-sectional imaging of the thorax were also excluded. Informed consent was obtained from all the participants or their parents/legal guardian for minors (under the age of 18). A subset of the current study population was previously reported by Hannoush et al. and Jamis-Dow et al.^17,18^.

### Participant imaging

Individuals referred to our institution for Proteus syndrome underwent baseline cardiothoracic imaging of the thorax to assess the degree of cardiothoracic involvement. Subsequent follow-up exams were also obtained if individuals became symptomatic (e.g. CTA for pulmonary embolism). CT and cardiac MRI scanner details are in Supplemental Table S1. The cardiac MRI protocol included cine images in the three long axes and a volumetric short axis cine stack for anatomic and functional analysis. Cardiac optimized, multi-echo Dixon fat–water separation images were also obtained at the same slices as the cine images as previously described^19,20^. This optimized fat–water sequence was used for easy detection of intramyocardial fat, even in thin structures such as the right ventricular (RV) free wall. Gadolinium was administered in only three of the cardiac MR studies and late gadolinium enhancement images were obtained in each of them.

### Image analysis

All chest CT studies were evaluated on a PACS workstation (CareStream, Version 12.1.6.1005, Carestream Health, NY) independently by two readers who were fellowship-trained in thoracic and body imaging and who were aware of the Proteus syndrome diagnosis but were blinded to all other clinical information. The readers (A.S., 10 years of experience and E.B.T., 13 years of experience) evaluated the lungs, heart, pleura, thoracic vasculature, chest wall and mediastinum for cardiothoracic imaging findings that were previously reported in the literature (Supplemental Table S2)^7,12,14–18,21–23^. The entire thorax was assessed for the presence of nodules or masses suspicious for malignancy^8,17^ and any other additional thoracic findings. Malignancy was determined via biopsy of suspicious lesions.

Two fellowship-trained cardiac MRI readers (A.S., 10 years of experience and A.J.B., 3 years of experience) independently reviewed the cardiac MRI studies. Global and regional cardiac function were assessed using cardiac MR cine images. Functional cardiac MRI data (left ventricular ejection fraction (LVEF), left ventricular (LV) end-diastolic volume, LV end-diastolic volume index, LV end-systolic volume, LV end-systolic volume index, LV stroke volume, LV stroke volume index, cardiac output, cardiac index, anteroseptal wall thickness and posterolateral wall thickness) was analyzed using postprocessing software (Argus, Siemens Healthineers, Erlangen, Germany). The presence, location, and distribution of intramyocardial fat was evaluated on cardiac fat–water separation images using the 17 segment model of the American Heart Association^24^. Segmental findings were combined into the distribution of LV walls: anterior, septal, inferior and lateral. The presence of fat was described as epicardial (outer portion of the wall), endocardial (inner portion of the wall), midwall (middle of the wall), transmural (full thickness involvement of the wall), diffuse (entire wall involved), or focal (small, < 5 mm area of a wall). Consensus read was performed to resolve any disagreements between the readers for both chest CT and cardiac MRI studies.

### Statistical analysis

Statistical analyses were performed using GraphPad Prism version 8.4.1 (676) for Windows (GraphPad Software, San Diego, California USA, http://www.graphpad.com). The Shapiro–Wilk test was used to assess the normality of the data. Percentage was used as the descriptive index for the qualitative variables and mean ± standard deviation (SD) were used to describe the quantitative variables. Differences in cardiac functional parameters between subjects with and without intramyocardial fat were compared with the unpaired t-test and nonparametric tests, as appropriate. Fisher’s exact test was used to compare the difference in the presence of fat location in LV and RV. A two-tailed *P* < 0.05 was considered to indicate a significant difference.

## Results

Of 53 individuals referred to our institution, 53 were diagnosed with Proteus syndrome and no individuals were diagnosed with AKT1-related overgrowth spectrum. Of these, 15 of the 53 individuals with Proteus syndrome did not have any cross-sectional cardiothoracic imaging. Thus, the remaining 38 individuals with Proteus syndrome (mean age: 23; range: 9–61 years) who had cross-sectional cardiothoracic imaging available for review were included in the study. This group comprised 23 males (mean age: 24 years; range: 9–61 years) and 15 females (mean age: 23 years; range: 10–54 years).

Of the 38 individuals with cross-sectional cardiothoracic imaging, 18 underwent only chest CT imaging, 16 chest CT imaging and cardiac MRI, and four only cardiac MRI. Thus, of the 38 individuals with cardiothoracic imaging, 34 had chest CT imaging and 20 had cardiac MRI. Among the 34 individuals who underwent chest CT, ten had a routine chest CT without contrast, four had a routine chest CT with contrast and 20 had CT pulmonary angiography.

### Cardiac involvement

**Figure 1:**
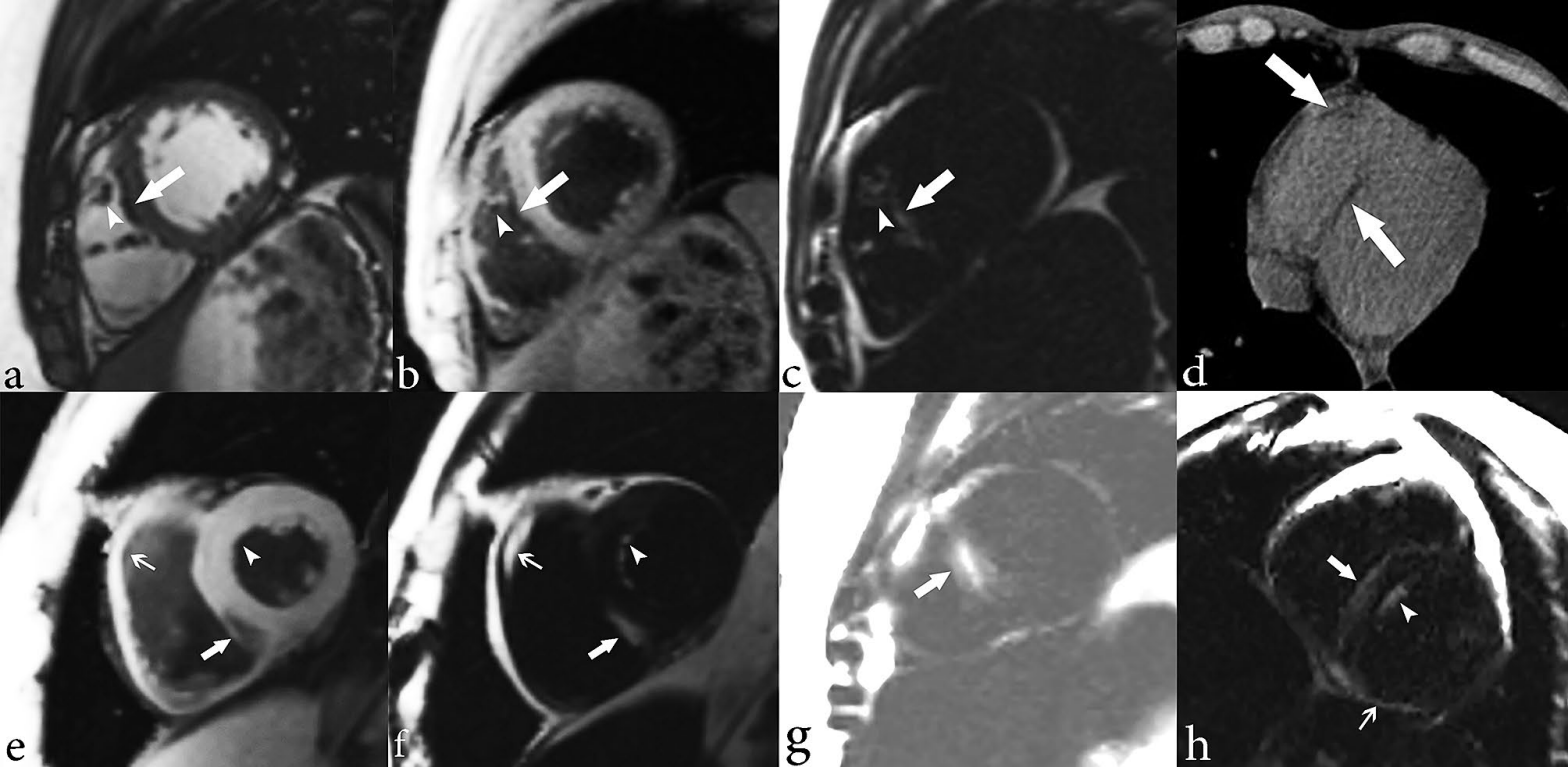
Cardiac MR images depicting intramyocardial fat in a 21-year-old female with Proteus syndrome. (**a**) Short axis cine image of a midventricular slice shows some areas of low signal intensity within the right ventricular (RV) trabeculae (arrowhead) and RV side of the interventricular septum (arrow) secondary to areas of chemical shift artifact caused by the presence of small areas of fat in the myocardium. Short-axis cardiac optimized multi-echo Dixon water (**b**) and fat (**c**) separation images of a mid-ventricular slice. (**b**) Short axis water image of the mid heart shows areas of intramyocardial fat as low signal intensity, for example within the RV trabeculae (arrowhead) and along the RV side of the interventricular septum (arrow). The fat image (**c**) shows areas of high signal intensity compatible with fat within the RV trabeculae (arrowhead) and RV endocardial side of the interventricular septum (arrow). (**d**) CT image of the chest confirms areas of fat within the RV side of the interventricular septum and right ventricular free wall of the heart (arrows). Patterns of intramyocardial fat are depicted on (**e**–**g**). Short axis water image (**e**) of a mid LV slice shows areas of midwall (thick arrow) and subendocardial (arrowhead) fat in the left ventricle as areas of low signal intensity. There is also fat involving the RV free wall (thin arrow). A corresponding fat image (**f**) shows the midwall (thick arrow), subendocardial (arrowhead), and RV wall fat (thin arrow) as areas of high signal intensity. Note that the subendocardial fat which is difficult to appreciate on the water image because of its similar signal intensity to the blood pool is very obvious on the fat image. (**g**) Short axis fat image of the mid LV shows a transmural area of high signal intensity corresponding to transmural fat within the septal wall (arrow). (**h**) Short axis fat image of a mid LV slice shows areas of fat as high signal intensity in several distributions: epicardial in the inferior wall (thin arrow), endocardial on the RV side of the interventricular septum (thick arrow) and endocardial on the LV side of septal wall (arrowhead).

Of the cohort of 38 patients who underwent imaging of the thorax, a subset of 20 individuals underwent dedicated cardiac MRI assessment for myocardial fat. Nine of the 20 individuals (45%) showed intramyocardial fat on the fat–water separation sequence (Fig. 1). Within the LV, the intramyocardial fat was more commonly present in the septal wall (n = 9, 45%) compared to other walls of the LV (*P* = 0.0029). The RV was involved less often than the LV (n = 6 and n = 9, respectively). Within the RV, the RV free wall was the most common location for the intramyocardial fat (n = 6, 30%), however this was not significantly different from other RV locations (*P* > 0.05). Intramyocardial fat distribution patterns were variable and included endocardial, midwall, epicardial, transmural, diffuse, and focal (Table 2). Some individuals had more than one pattern of intramyocardial fat within the same LV segment. In the LV, intramyocardial fat was most commonly located in an endocardial distribution. In the RV, intramyocardial fat most commonly was present diffusely within the RV free wall.

**Table 2:** The location and distribution of intramyocardial fat in a subset of 20 individuals with Proteus syndrome who underwent cardiac MRI evaluation.

Intramyocardial fat	No. of individuals (%)	Pattern of distribution
**LV involvement**		
LV walls		
Anterior	3 (15)	Endocardial n = 1, midwall n = 1, diffuse n = 1
Septal	9 (45)	Endocardial n = 7, midwall n = 2, transmural n = 2, focal n = 1
Inferior	2 (10)	Endocardial n = 2, epicardial n = 1
Lateral	3 (15)	Endocardial n = 2, diffuse n = 1
Apex	0 (0)	
Other LV locations		
Papillary muscles	3 (15)	
LV trabeculae	3 (15)	
**RV involvement**		
RV free wall	6 (30)	Diffuse n = 6
Moderator band	4 (20)	
RV trabeculae	3 (15)	

RV = right ventricle, LV = left ventricle.

Chest CTs were obtained in 34 individuals and also evaluated for the presence of intramyocardial fat (Fig. 1, Table 4). 16 of the 34 individuals, which included seven of the nine individuals with intramyocardial fat on cardiac MR, also had a chest CT. However, intramyocardial fat was missed on CT in one of these seven individuals by both readers who agreed this was most likely due to cardiac motion on the non-gated chest CT study. There were a total of 18 patients who only had chest CTs and no dedicated cardiac MRI to assess for intramyocardial fat. Evaluation of these 18 individuals who only had chest CTs for intramyocardial fat only detected one individual with intramyocardial fat. Thus, evaluation of the chest 34 chest CTs only detected a total of 7 (21%) cases of intramyocardial fat.

There was no statistically significant difference among any of the cardiac functional parameters in the individuals with intramyocardial fat compared to those without (Table 3). The average LVEF of the individuals who underwent cardiac MR was normal (60 ± 5) with only one individual (female, age = 10 years-old) who had intramyocardial fat that was associated with a mildly decreased LVEF of 53% and mild diffuse hypokinesis without a focal regional wall motion abnormality. No wall motion abnormalities were found in any of the other individuals. Only three individuals received gadolinium and only one individual had a small patch of subendocardial late gadolinium enhancement in the apical lateral wall, however there was no associated wall motion abnormality or decreased function.

**Table 3:** Functional cardiac MRI information from a subset of 20 individuals with Proteus syndrome who underwent cardiac MRI.

Parameter (units)	Individuals with fat (n = 9)		Individuals without fat (n = 11)		P-value
	Range	Mean ± SD	Range	Mean ± SD	
LVEF (%)	53–69	60 ± 5	55–67	61 ± 4	0.56
LVEDV (ml)	64–245	132 ± 50	75–228	140 ± 42	0.72
LVESV (ml)	20–111	55 ± 26	25–103	55 ± 20	0.99
LVSV (ml)	44–134	77 ± 25	46–125	85 ± 23	0.50
CO (ml/min)	4–9	6 ± 1.5	4–8	6 ± 1	0.77
LVEDVI (ml/m^2^)	60–123	83 ± 19	63–121	84 ± 17	0.90
LVESVI (ml/m^2^)	21–49	33.5 ± 8	22–55	33 ± 10	0.93
LVSVI (ml/m^2^)	34–80	50 ± 13	38–66	51 ± 8	0.81
CI (ml/min/m^2^)	2–8.5	4 ± 2	3–5	4 ± 1	0.82
AS (mm)	4–13	7 ± 3	4–9	6 ± 1	0.30
PL (mm)	4–10	6 ± 1.5	3–7	6 ± 1	0.94

LVEF = left ventricular ejection fraction, LVEDV = left ventricular end-diastolic volume, LVESV = left ventricular end-systolic volume, LVSV = left ventricular stroke volume, CO = cardiac output, LVEDVI = left ventricular end-diastolic volume index, LVESVI = left ventricular end-systolic volume index, LVSVI = left ventricular stroke volume index, CI = cardiac index, AS = anteroseptal wall thickness, PL = posterolateral wall thickness.

### Airway and lung parenchymal involvement

**Figure 2:**
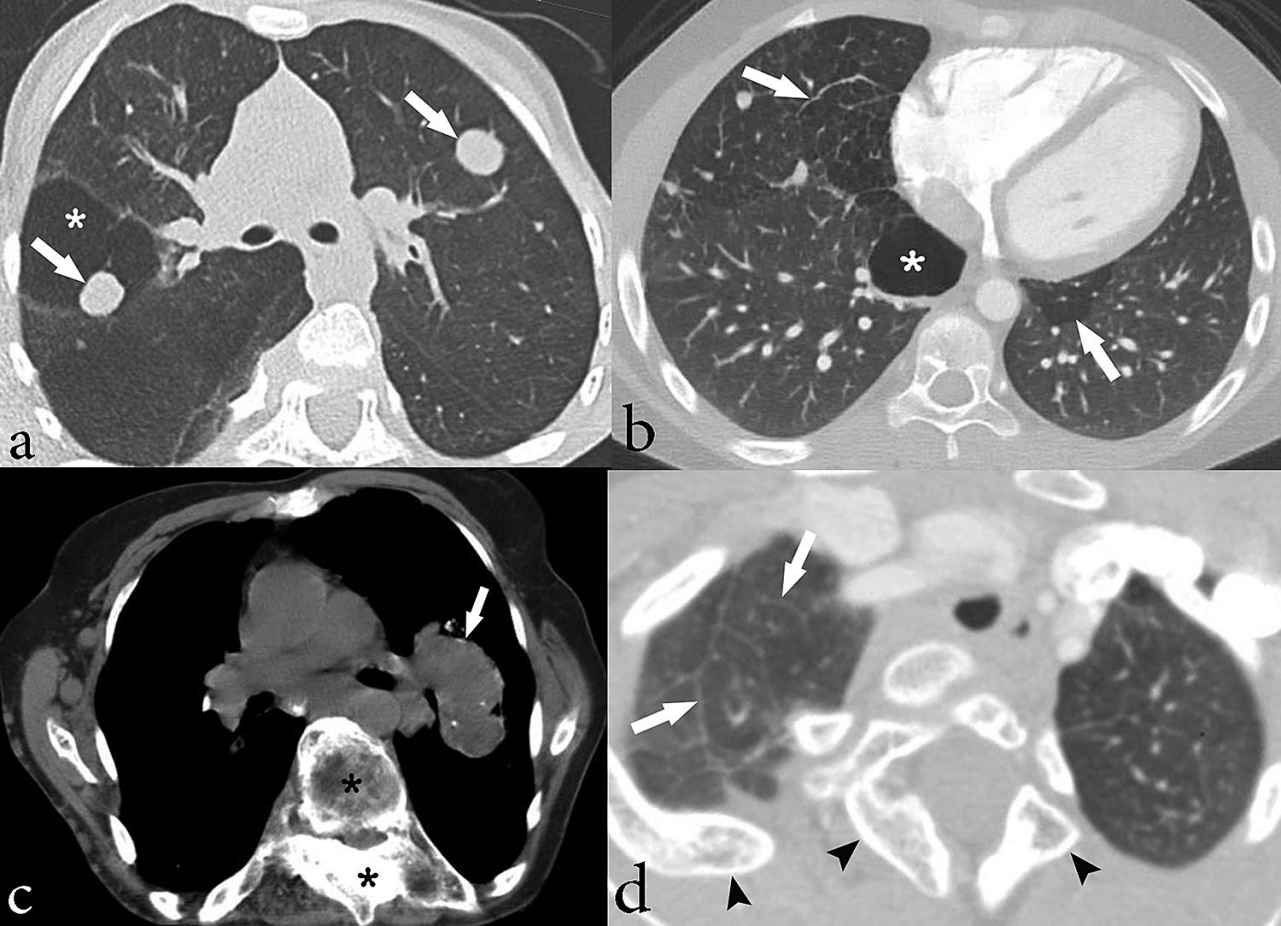
Chest CT images showing lung parenchymal involvement in individuals with Proteus syndrome. Axial CT images in lung windows show (**a**) nodules in the bilateral upper lobes measuring up to 17 mm in size (arrows) in a 9-year-old male. An area of cystic change is also noted in the right upper lobe (asterisk); (**b**) areas of hyperlucent lung parenchyma within the right middle lobe and left lower lobe (arrows) in a 10-year-old male. There is also a cyst in the right lower lobe (asterisk) that abuts a curvilinear band of scarring posteriorly. (**c**) Axial CT image in soft tissue windows shows a lobulated mass in the perihilar left upper lobe (arrow) in a 38-year-old female nonsmoker, which was found to be primary lung adenocarcinoma on pathology. Overgrowth of the vertebral body and posterior elements of the spine are also noted (asterisks). (**d**) Axial CT image in lung windows shows subtle interlobular septal thickening (arrows) in the right upper lobe in a 14-year-old female. Skeletal overgrowth involving the posterior spinous elements and right posterior rib are also noted (arrowheads).

**Figure 3:**
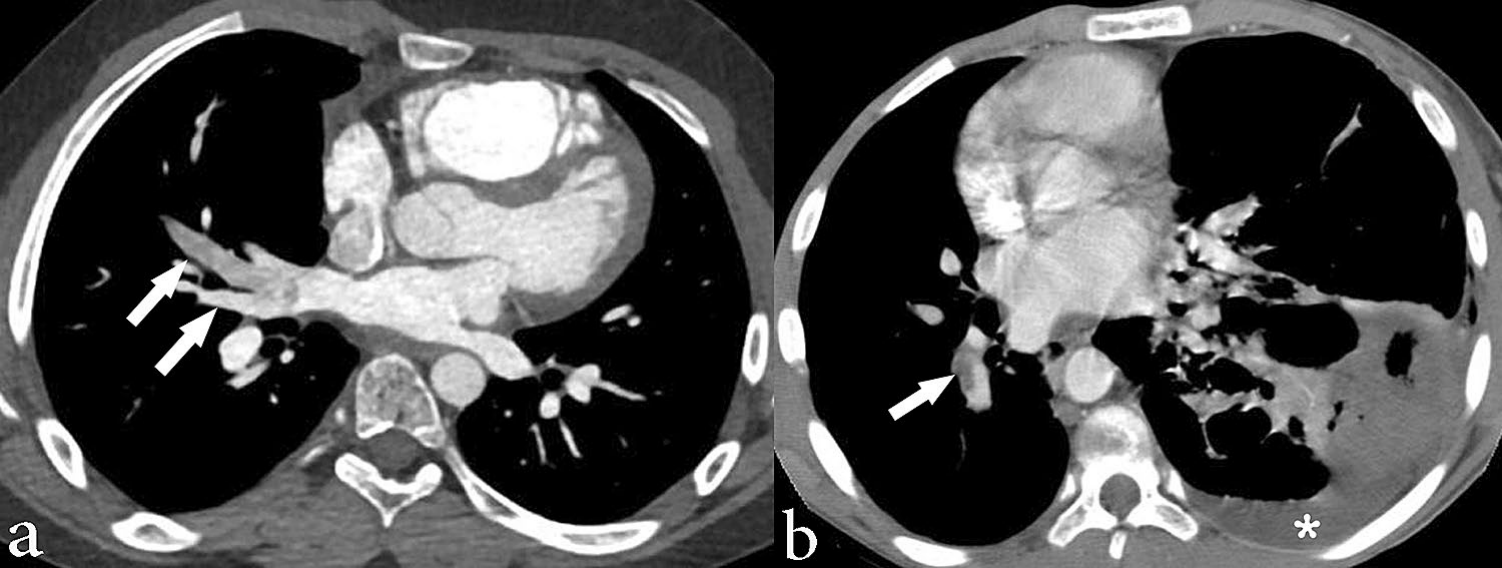
Chest CT images in soft tissue windows showing vascular abnormalities in individuals with Proteus syndrome. (**a**) Axial CT image shows abnormally enlarged right pulmonary veins (arrows) in a 15-year-old male. (**b**) Axial CT image shows a filling defect in the right lower lobe pulmonary artery (arrow) compatible with a pulmonary embolism in a 34-year-old male. Incidentally, there is also consolidation secondary to pneumonia within the left lower lobe as well as an associated small left pleural effusion (asterisk).

Among those who underwent chest CT (n = 34), the most common findings in the lung parenchyma were hyperlucent lung parenchyma (n = 17, 50%), pulmonary nodules (n = 17, 50%), and bandlike areas of scarring (n = 19, 56%). Hyperlucent lung parenchyma usually involved < 50% of the lobe and was most common in the right upper lobe (n = 11, 32%). Lung cysts nearly always involved < 50% of a lobe and were most commonly involved the right lower lobe (n = 6, 18%). Both calcified and non-calcified nodules were present. Nine individuals had only non-calcified nodules, four individuals had only calcified nodules and four had both calcified and non-calcified nodules. All nodules were well-circumscribed, smoothly marginated, and round or oval in shape. The calcified nodules were completely calcified and ranged in size from 1 to 6.5 mm. Non-calcified nodules ranged from 1 to 17 mm in size and were completely soft tissue in attenuation. Large non-calcified nodules (10 to 17 mm) were observed in only one individual of our cohort. Interlobular septal thickening was present in four individuals (12%). Incidental, acute lung parenchymal findings in 4 individuals (12%) who had pneumonia at the time of scanning included consolidation and ground glass opacity. Minimal bronchiectasis was present seen in one individual (3%). Tracheal diverticulum and tracheal bronchus were incidental findings in two individuals (6%). One individual (38 year-old non-smoker) had a perihilar, left upper lobe mass (3%), which was biopsied and findings were compatible with primary lung adenocarcinoma. These findings are illustrated in Figs. 2 and 3 and summarized in Table 4.

**Table 4:** Summary of characteristic cardiothoracic imaging findings of a population of 34 individuals with Proteus syndrome who underwent chest CT imaging.

Cardiothoracic involvement				No. of individuals	Percentage
Pulmonary and airway involvement	Hyperlucent lung parenchyma	RUL	< 50%	7	32
			≥ 50%	4	
		RML	< 50%	2	15
			≥ 50%	3	
		RLL	< 50%	6	24
			≥ 50%	2	
		LUL	< 50%	5	26
			≥ 50%	4	
		Lingula	< 50%	0	9
			≥ 50%	3	
		LLL	< 50%	5	26
			≥ 50%	4	
	Cysts	RUL	< 50%	1	3
			≥ 50%	0	
		RML	< 50%	1	3
			≥ 50%	0	
		RLL	< 50%	6	18
			≥ 50%	0	
		LUL	< 50%	4	12
			≥ 50%	0	
		Lingula	< 50%	0	3
			≥ 50%	1	
		LLL	< 50%	1	9
			≥ 50%	2	
	Nodule			17	50
	Band-like areas of parenchymal scarring			19	56
	Interlobular septal thickening			4	12
	Bronchiectasis			1	3
Pleural involvement	Thickening			5	15
Cardiac involvement	Intramyocardial fat			7	21
Chest wall abnormalities	Rib overgrowth			19	56
	Scapula overgrowth			1	3
	Fat overgrowth			10	29
	Fatty infiltration of paraspinal muscle			3	9
	Spinal abnormalities	Vertebral body overgrowth and asymmetric height		26	76
		Posterior elements of the spine overgrowth	Spinous process	15	44
			Lamina	23	68
			Transverse process	23	68
		Scoliosis		32	94
	Lipoma			1	3
	Asymmetric breast enlargement			3	9
Vascular involvement	Vascular anomaly			0	0
	Systemic venous dilation			10	29
	Pulmonary venous dilation	Right upper		11	62
		Right lower		11	
		Left upper		7	
		Left lower		14	
	Thromboembolism			2	6
	Aortic aneurysm			2	6
Mediastinal involvement	Large thymus			1	3
	Mediastinal lipomatosis			1	3
	Lymphadenopathy			3	9
Malignancy	Adenocarcinoma			1	3

RUL = right upper lobe, RML = right middle lobe, RLL = right lower lobe, LUL = left upper lobe, LLL = left lower lobe.

### Vascular involvement

More than half of the individuals with chest CTs had pulmonary venous dilation (n = 21, 62%), as shown in Fig. 3. Affected pulmonary veins were diffusely dilated. Right and left pulmonary veins were involved similarly (n = 17 and n = 16, respectively). Ten individuals also had abnormal enlargement of systemic veins: internal jugular vein, subclavian vein, brachiocephalic vein, superior vena cava and the azygos vein. Pulmonary embolism was detected in two symptomatic individuals (6%) on CT angiography (Fig. 3). Mild aneurysmal dilation (diameter 42–43 mm) of the ascending aorta was present in two individuals (6%). The vascular findings are summarized in Table 4.

### Pleural involvement

Pleural effusion and thickening were detected in three (9%) and five (15%) individuals, respectively (Table 4). Pleural effusion was found incidentally in individuals acutely ill with pneumonia and likely unrelated to Proteus syndrome. No individuals had pleural nodules.

### Mediastinal involvement

**Figure 4:**
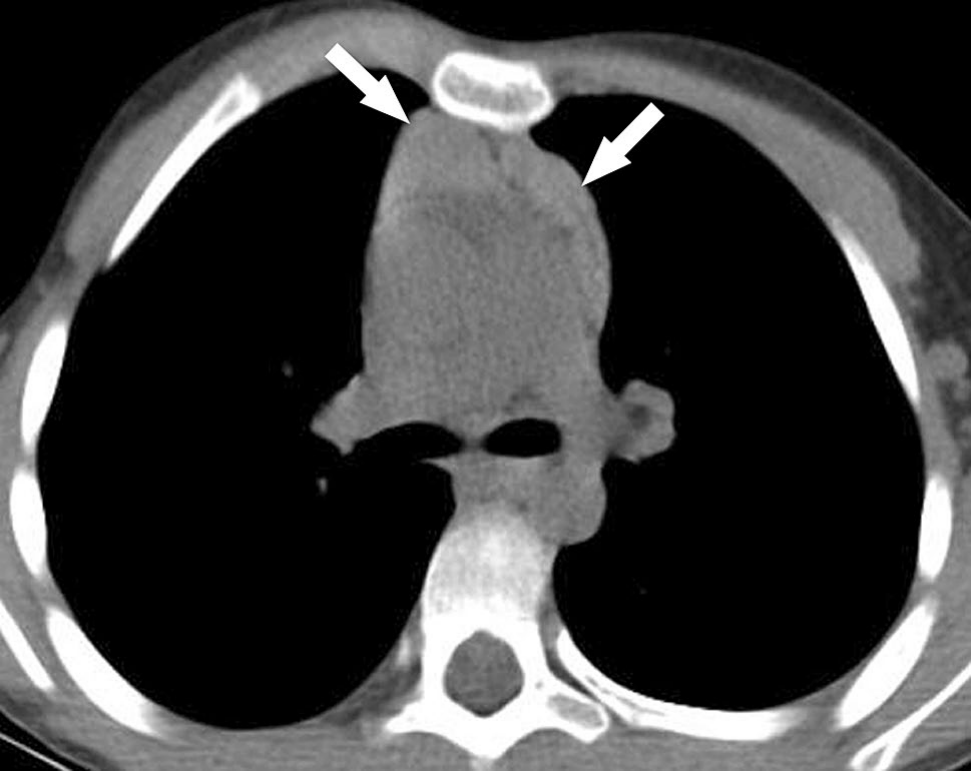
Axial chest CT image in soft tissue windows in a 20-year-old male individual with Proteus syndrome who was otherwise healthy shows an enlarged thymus relative to the individual’s age (arrows).

Mediastinal lipomatosis (n = 1, 3%) and enlargement of the thymus (Fig. 4) relative to age (n = 1, 3%) were uncommon. Mediastinal findings are summarized in Table 4.

### Chest wall involvement

**Figure 5:**
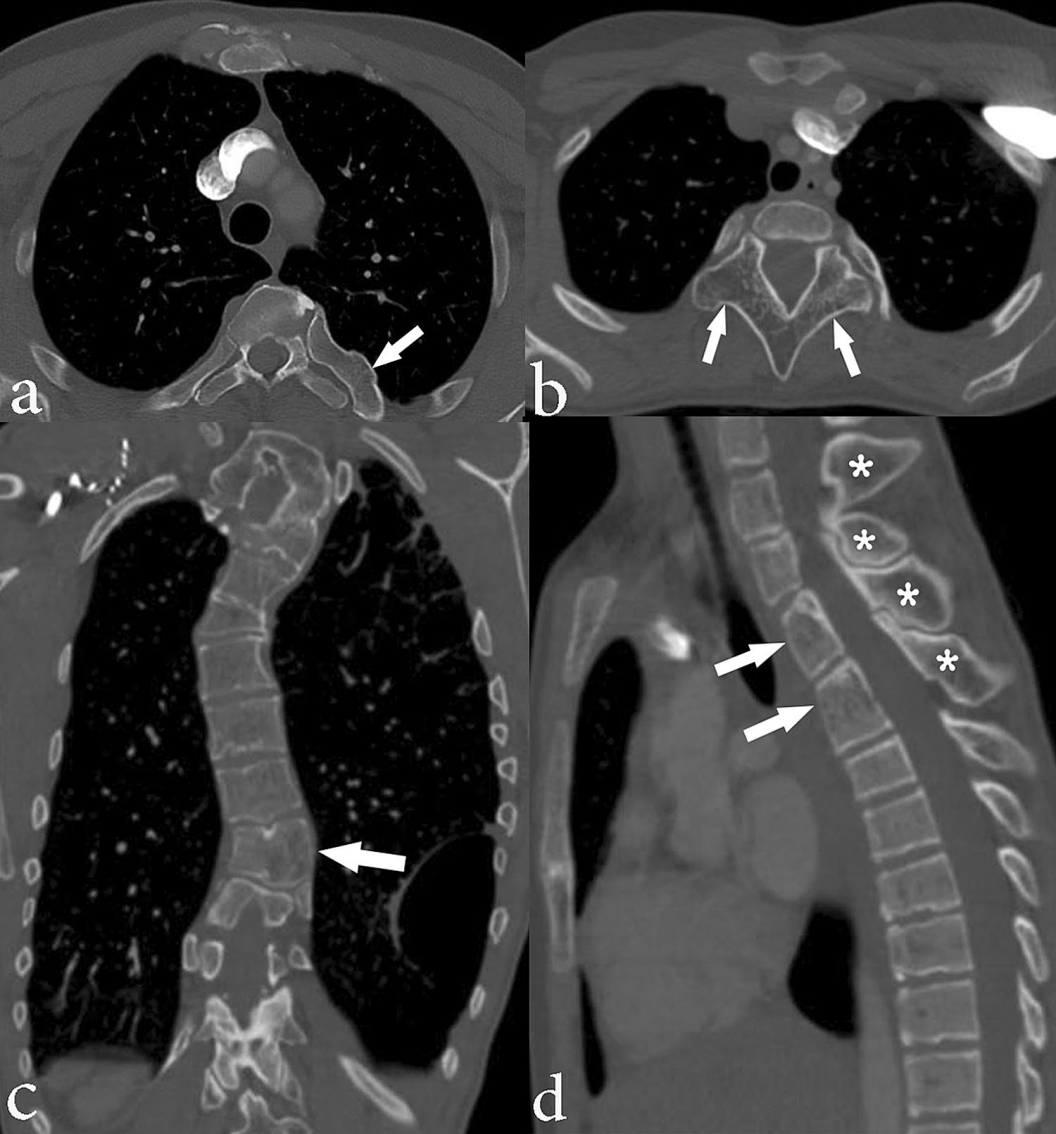
Chest CT images depicting skeletal abnormalities in individuals with Proteus syndrome. Axial CT image of the chest in bone windows shows (**a**) overgrowth of the left fifth posterior rib (arrow) in a 61-year-old male (note normal size of the contralateral right posterior fifth rib) and (**b**) overgrowth of the posterior elements of the spine (arrows) in a 22-year-old female. (**c**) Coronal CT image in bone windows in a 36-year-old male shows scoliosis of the thoracic spine. There is asymmetric overgrowth of multiple vertebral bodies resulting in asymmetric vertebral body heights (example depicted by the arrow). (**d**) Sagittal CT image of the spine in bone windows in a 22-year-old female shows overgrowth of several spinous processes (asterisks) and several upper vertebral bodies (arrows).

**Figure 6:**
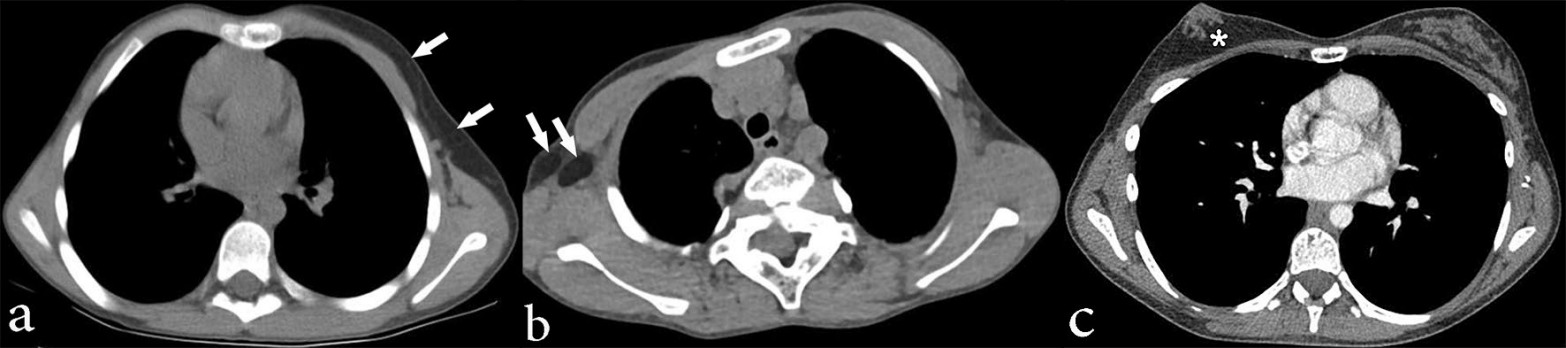
Axial chest CT images in soft tissue windows depict abnormal adipose overgrowth in individuals with Proteus Syndrome. Axial CT images show (**a**) asymmetric fat overgrowth in the left chest wall (arrows) in a 20-year-old male; (**b**) well-circumscribed areas of fat likely represent lipomas within the right axilla (arrows) in a 9-year-old male; (**c**) asymmetric breast tissue in a 22-year-old female. There is a paucity of breast tissue in the right breast with an increased amount of adipose tissue (asterisk).

Skeletal findings included scoliosis, asymmetric vertebral body growth, overgrowth of posterior elements of the spine, ribs, and scapulae. Scoliosis secondary to asymmetric overgrowth of various portions of the spine was the most common chest wall manifestation (n = 32, 94%) in this study. Skeletal findings of Proteus syndrome are depicted in Fig. 5. Overgrowth of chest wall fat was seen in ten individuals (29%). One individual (3%) had a well-circumscribed area of focal fat overgrowth in the right axilla suspicious for lipoma and three individuals (9%) had asymmetric breast size and/or asymmetric breast tissue/fat composition related to fat overgrowth. In individuals who had both deformed skeletal anatomy (e.g. scoliosis) as well as soft tissue asymmetry, the soft tissue asymmetry was not associated with the areas of skeletal deformity. Chest wall adipose overgrowth findings are depicted in Fig. 6 and summarized in Table 4.

In summary, nearly all of the individuals in our cohort (n = 37, 97%) had cardiothoracic involvement of Proteus syndrome. On chest CT, skeletal involvement was the most common (n = 32, 94%) followed by airway and lung parenchymal involvement (n = 29, 85%), vascular involvement (n = 23, 68%), pleural involvement (n = 5, 15%), and mediastinal involvement (n = 3, 9%). On cardiac MRI, intramyocardial fat was common (n = 9, 45%). The majority of individuals (n = 29, 85%) had both musculoskeletal and pulmonary involvement. Approximately a third of the cohort (n = 12, 35%) had involvement of musculoskeletal, pulmonary and vasculature structures within the thorax.

## Discussion

In this study, we were able to further define the prevalence of cardiothoracic imaging findings in Proteus syndrome. Intramyocardial fat was common (45%) on cardiac MRI and its severity had no association with age. Although present in many places in both ventricles, the intramyocardial fat was always present in the septal wall. This intramyocardial septal wall fat is on the mild end of a spectrum of abnormal fat growth that includes the myocardial septal lipoma described within the clinical diagnostic criteria for Proteus syndrome (Table 1) at the more severe end of the spectrum. Interestingly, intramyocardial fat was also detected on the routine, non-cardiac gated chest CTs about half of the time (21%). This illustrates the importance of looking at the heart on routine chest CT exams, but also confirms that chest CT is an imperfect technique for intramyocardial fat detection as it missed cases detected by cardiac MRI. The intramyocardial fat does not appear to correlate with wall motion abnormalities or significantly impact LV function. Although a previous study reported arrhythmia (right bundle branch block) associated with intramyocardial fat in Proteus syndrome^25^, we observed no arrythmia associated with the presence of intramyocardial fat or any other adverse cardiac complications.

As Proteus syndrome is an overgrowth syndrome, we hypothesize that this intramyocardial fat may represent overgrowth of areas of physiologic intramyocardial fat that has been well described in healthy individuals. In healthy individuals, intramyocardial fat is commonly seen in the RV up to 85% of the time, but is only present in the LV or in both ventricles < 20% of the time^26,27^. Thus, Proteus syndrome differs by commonly having intramyocardial fat in both ventricles, with LV predominance over the RV. However, intramyocardial fat in Proteus syndrome is associated with no wall motion abnormality, a feature it shares with the intramyocardial fat present in healthy individuals. Unlike Proteus syndrome, pathologic conditions with intramyocardial fat tend to have associated ventricular chamber enlargement, dysfunction and wall motion abnormalities. These include arrhythmogenic RV cardiomyopathy (ARVC), healed myocardial infarction, dilated cardiomyopathy, and Duchenne’s muscular dystrophy. It should be noted that myocardial fat is no longer part of the diagnostic criteria for ARVC^28^. Further follow-up of the individuals with intramyocardial fat will be necessary to characterize its behavior over time and determine if it behaves similarly to a hamartomatous anomaly or more similarly to a fatty neoplasia.

Pulmonary nodules, areas of hyperlucent lung, and bandlike areas of scarring were present in at least 50% of individuals, which is much greater than the 8–13% described in prior reports^8,12,17,29^. Although we did not have pathology available for the pulmonary nodules in this study, they are suspected to be hamartomas based on prior published pathology^12^. In regards to the hyperlucent lung parenchyma, previous studies with pathology found that the areas of hyperlucent lung parenchyma corresponded to panlobular emphysema^12,15^. However, additional pathological evaluation is needed to further validate this correlation.

Pulmonary venous dilation was present in over half of our cohort. However, systemic venous dilation was less common. Venous dilation in Proteus syndrome is secondary to vascular overgrowth, but it remains uncertain as to why the pulmonary veins are affected more commonly than systemic veins. Vascular anomalies (tumors, malformations) have been previously reported^6,8,30^, however we did not observe any.

Chest wall abnormalities related to dysregulated skeletal and fat overgrowth are extremely common in Proteus syndrome^1,5,7,17^, and were also commonly observed in the current study. Scoliosis secondary to overgrowth of various vertebral elements is almost universally present and tends to be progressive in Proteus syndrome, leading to significant deformity of the chest wall and eventual respiratory compromise that may necessitate orthopedic intervention. Additionally, we observed significant asymmetric breast size and/or composition related to an abnormal ratio of fat and parenchymal tissue in the affected breast. To our knowledge, this finding has not been previously reported.

The main thoracic complications of Proteus syndrome include pulmonary thromboembolism, respiratory failure, pneumonia, and malignancy. Pulmonary thromboembolism in Proteus syndrome, which was observed in our cohort, is elevated relative to the general population and is thought to be due to decreased anticoagulation proteins, venous stasis in vascular malformations, as well as the effects of the pathogenic AKT1 variant on endothelial cells^1–3,5,8,10^. Proteus syndrome is associated with an increased risk of developing malignancy of any kind which is already well documented in the literature, with meningioma and ovarian cystadenoma being among the more common^1,2,6–8^. In our population of individuals with Proteus syndrome, we found one left upper lobe mass that was found to be primary lung adenocarcinoma in an individual with no other risk factors other than Proteus syndrome.

## Limitations

Our study has several limitations. The main limitation is that it is retrospective in nature. Although we have a large cohort of individuals for such a rare disease, not all individuals had both cardiac MRI and chest CT imaging performed. Second, the majority of the cardiac MRI studies and several of the chest CTs were performed without contrast. Lack of contrast limited our ability to assess for myocardial fibrosis on cardiac MRI and to assess for pulmonary embolism on the non-contrast chest CTs. Third, although many individuals in our cohort had lung parenchymal findings, pulmonary function testing was not available for correlation. This is an important clinical question and will be looked at in a future study. Finally, pathology was not available for all of the imaging findings we observed.

## Conclusion

Proteus syndrome is a rare disorder with many cardiothoracic imaging findings that are much more common than previously described in the literature. The most common areas of involvement include the chest wall (skeletal and adipose tissue), lung parenchyma, thoracic vasculature, and myocardium. Additionally, it is already known that this syndrome has an increased risk of complications that include pulmonary thromboembolism, respiratory failure, pneumonia, and malignancy—findings which we also observed in our cohort of individuals with Proteus syndrome.

## Supplementary Information

Supplementary Information.
