# Supplementary material for: Cardiothoracic imaging findings of Proteus syndrome
Source: Sci Rep. 2021 Mar 22;11:6577. doi: 10.1038/s41598-021-86029-0 (PMC7985501; doi:10.1038/s41598-021-86029-0)
Supplement: Supplementary file 1 — Supplementary Information. [file 41598_2021_86029_MOESM1_ESM.docx]

**Cardiothoracic Imaging Findings of Proteus Syndrome**

S. Mojdeh Mirmomen^1^, M.D.; Andrew E. Arai^1^, M.D.; Evrim B. Turkbey^2^, M.D.; Andrew J. Bradley^1^, M.D., Julie C. Sapp^3^,Sc.M., C.G.C.; Leslie G. Biesecker^3^, M.D., Arlene Sirajuddin^1*^, M.D.

^1^Cardiovascular and Pulmonary Branch, National Heart Lung and Blood Institute, National Institutes of Health, Building 10, Room B1D416, 10 Center Drive, Bethesda, Maryland 20814

^2^Radiology and Imaging Sciences, National Institutes of Health, Building 10, Room 1C336, Bethesda, Maryland 20814

^3^Genetic Disease Research Branch, National Human Genome Research Institute, National Institutes of Health, Building 10, Room 8D47E, Bethesda, Maryland 20814

*Arlene Sirajuddin, M.D.

Building 10, Room B1D416

10 Center Drive

Bethesda, MD 20814

National Heart, Lung, and Blood Institute

National Institutes of Health

Email: [arlene.sirajuddin@nih.gov](mailto:arlene.sirajuddin@nih.gov)

Tel: +1 301-594-3475

Fax: +1 301-896-7521

Supplemental Table S1 Detailed scanner information for imaging studies obtained in our cohort.

| Imaging modality | Scanner type |
| --- | --- |
| Routine chest CT (n=14) | GE Medical Systems LightSpeed Ultra n=2, GE Medical Systems LightSpeed QX/I n=2, Siemens Biograph 128 n=1, Siemens Definition n=1, Siemens Somatom Force n=4, Philips iCT 256 n=2, Philips Brilliance 64 n=2 |
| CT pulmonary angiography (n=20) | Siemens Somatom Force n=7, Siemens Biograph 128 n=1, Siemens Definition n=8, Siemens Somatom Definition Flash n=2, GE Medical Systems LightSpeed Ultra n=1, GE Medical Systems Discovery CT750 HD n=1 |
| Cardiac MRI (n=20) | Siemens 3T Skyra n=19, Siemens 1.5T Aera n=1 |

Supplemental Table S2 Cardiothoracic imaging findings of Proteus syndrome based on prior literature.

| Lungs | Hyperlucent lung parenchyma^1^, cyst^1^, nodule, fibrosis, interlobular septal thickening, bronchial and tracheal abnormalities |
| --- | --- |
| Heart | Intramyocardial fat |
| Pleura | Nodules and thickening |
| Thoracic vasculature | Vascular anomaly, systemic^2^ and pulmonary venous dilation^3^, arterial abnormalities, aortic dilation^3^, and pulmonary embolism |
| Chest wall | Skeletal^4^, adipose^4^, and muscular abnormalities. |
| Mediastinum | Thymic enlargement^5^ and mediastinal lipomatosis |
| Malignancy | Assessed for the presence of nodules or masses suspicious for malignancy |

Hyperlucent lung parenchyma was defined as an area of lung on CT with a lower density than surrounding normal lung at full inspiration. Lung cyst was defined as a well-defined air-filled lucency with a thin (1mm or less) wall. The severity of hyperlucent lung parenchyma and cysts was assessed for each lobe, and described as ˂50% or ≥50% of the involved lobe.

^2^ Systemic venous dilation was qualitatively assessed for grossly dilated systemic venous structures (i.e. azygous vein equal to or greater than the size of the aorta).

^3^ Aortic dilation and pulmonary venous dilation were measured using previously described criteria by Litmanovitch et al and Kim Y-H et al respectively. Upper limit of normal aorta size used was 4 cm for the ascending aorta and 3 cm for the descending aorta. Pulmonary vein diameters were measured from a short-axis view of each pulmonary vein at the ostia and subsequently at 5 mm intervals from the ostia and compared to normal values for men and women as detailed in Kim Y-H et al [21-22].

^4^ Skeletal and adipose overgrowth was defined as asymmetric enlargement of the portions of the skeleton or areas of adipose tissue.

^5^ The thymus was measured in the following dimensions: anterior-posterior, craniocaudal, transverse. Thymic measurements were compared with normal measurements according to age per Francis IR et al [23]
